# Controllable stripping of radiolabeled group *in vivo* to optimize nuclear imaging *via* NO-responsive bioorthogonal cleavage reaction†

**DOI:** 10.1039/d0ra07186b

**Published:** 2020-11-03

**Authors:** Hua Li, Lumei Huang, Hailong Jiang, Jianyang Fang, Zhide Guo, Fei Gao, Mei Chen, Duo Xu, Zijing Li, Xianzhong Zhang

**Affiliations:** Center for Molecular Imaging and Translational Medicine, State Key Laboratory of Molecular Vaccinology and Molecular Diagnostics, School of Public Health, Xiamen University Xiamen Fujian 361102 China zhangxzh@xmu.edu.cn zijing.li@xmu.edu.cn; College of Materials Science and Engineering, Hunan University Changsha Hunan 410082 China

## Abstract

A novel “turn-off” strategy for controllable radionuclide clearance is established. 1,4-dihydropyridine (DHP) is used as a conditional linker to connect a radioisotope labeled moiety and nano-agent. A highly specific, sensitive and effective C–C bond cleavage of DHP happens *in vivo* when treated with nitric oxide which is provided by glyceryl trinitrate (GTN). The radioactive cut-off part from the nanoparticle is observed to be cleared quickly by microSPECT-CT. 3–5 times decreases of radioactivity in the blood, kidneys, intestine, heart and lungs are observed after GTN treatment in a biodistribution assay. The radioactivity redistribution indicates that the radioactive leaving part is indeed cut off and the radionuclide metabolism accelerated. Organ level internal dose assessment reveals the GTN treated groups carry only ½ the radiation dose of the control group. Collectively, a feasible pathway for controllable radionuclide clearance is for the first time provided for high contrast and low radiation nuclear imaging.

## Abstract 2

A novel “turn-off” strategy was developed for controllable radionuclide clearance in organisms.

## Graphical abstract

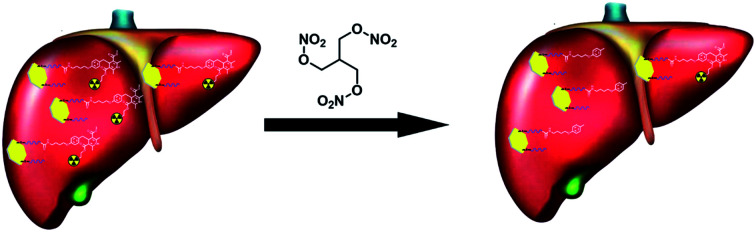

Radiolabeled compounds with biological activity, or radiotracers, have been widely used for nuclear imaging and radiation therapy. Compared with other imaging modalities, a unique problem in radiotracer-based imaging is that the radioactivity cannot be simply “turned off”. As a result, it is impossible to carry out multi-scans of the same organ with different tracers in a short period. For instance, there are three kinds of marker receptors (ER, PR, HER2) expressed in breast cancer. Different type of breast cancer expresses a different combination of these three. Therefore, three kinds of radiotracers that aim to bind the corresponding receptors will be used to distinguish individual breast cancer phenotype. The uptake of a second tracer can't be quantified accurately before the radioactivity of the first tracer decayed to background level, which may need one or two days depending on the half-life of radionuclide. So, it's necessary to develop a strategy to “quench” the radioactivity. Furthermore, nonspecific binding is inevitable, and the “noise” or “background” from non-specific binding of radiotracers to non-target proteins cannot be easily differentiated from the specific binding component.

Therefore, it's meaningful to develop a kind of reaction that could strip the radiation by control. To achieve this goal, the metabolism of the radioactive part should be accelerated. The biorthogonal cleavage reaction would perfectly meet the needs, if (1) cleavable reactions could happen by the control *in vivo*; (2) the cut-off part has a relatively fast metabolism; (3) radionuclide is linked on the cut-off part.^1^ One of the key issues that bio-orthogonal reactions resolve is to bind two components into one, and make the different metabolic rate of different components synchronized.^2^ As the reaction in the opposite direction of bio-orthogonal reaction, the bio-orthogonal cleavage reaction can extinguish the radiation by diversifying the metabolism of radionuclides and the slow metabolic targeting components.

Dihydropyridine (DHP) and its cleavage-triggering partner nitric oxide (NO) can perfectly meet the three conditions. NO, a hydrophobic signal molecule, could spread without any transmembrane transporter, which means NO could spread quickly and spend little energy.^3–5^ Many kinds of NO donor drugs are commercially available, such as glyceryl trinitrate (GTN), sodium nitroprusside, *etc.* In our previous work,^6^ it was demonstrated that the physiological concentration of NO is high enough to cleave the C–C bond of DHP. Furthermore, NO donor drugs could offer a circumstance with higher NO concentration than normal physiological concentration. Therefore, it is possible for the reaction between DHP and NO from donor drugs to occur *in vivo*. Herein, a NO-triggerred “turn-off” system to extinguish the radiation by cleaving the radionuclides from nanoparticles is present. Benzyl group substituted 1,4-dihydropyridine can be cleaved through controlled NO stimulation by intraperitoneal injection of GTN.

Nanoparticles as the major kind of theranostic agents have been developed quickly over these years.^7–11^ During the process of synthesis, nanoparticles with similar shape, scale and dispersion properties would be obtained by manipulating conditions precisely. Therefore, the metabolism of nanoparticles in the living body is more predictable than diverse small molecules. Especially, radionuclides labeled nanoparticles not only play roles in diagnosis but also work well on tumor therapy. Enhanced permeability and retention (EPR) effect make nanoparticles wonderful radiotherapy reagents.^12–15^ However, nanoparticles mean to be easily captured by the mononuclear phagocyte system (MPS), which makes the nanoparticles mostly enriched in liver besides in tumor.^16–20^ Furthermore, metabolism of nanoparticles are quite slow than most of the small molecules, which means the radionuclides labeled on nanoparticles have similar slow metabolism to their carriers. Unnecessarily loaded radiation from the nanoparticles will also harm normal liver cells where the nanoparticles accumulate heavily.

## Result and discussion

**Scheme 1:**
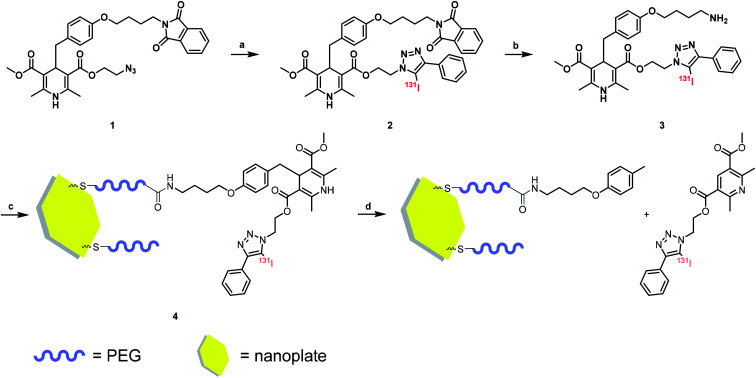
Radiolabeling procedure and method of nanoplate decoration. Reagent and condition: (a) CH_3_CN, [^131^I]I^−^, CuCl_2_, Et_3_N, 50 °C; (b) EtOH, N_2_H_4_·H_2_O, RT; (c) H_2_O, 4 °C; (d) NO solution.

A DHP based bifunctional intermediate (compound 1) was used as an activable linker to accomplish the “on-off” design. The azide group was labeled with ^131^I by a “click-like” reaction,^21^ and the phthalimide group was reduced to amino group by Gabriel reaction (Scheme 1) for conjugation of nanoplates.

**Fig. 1:**
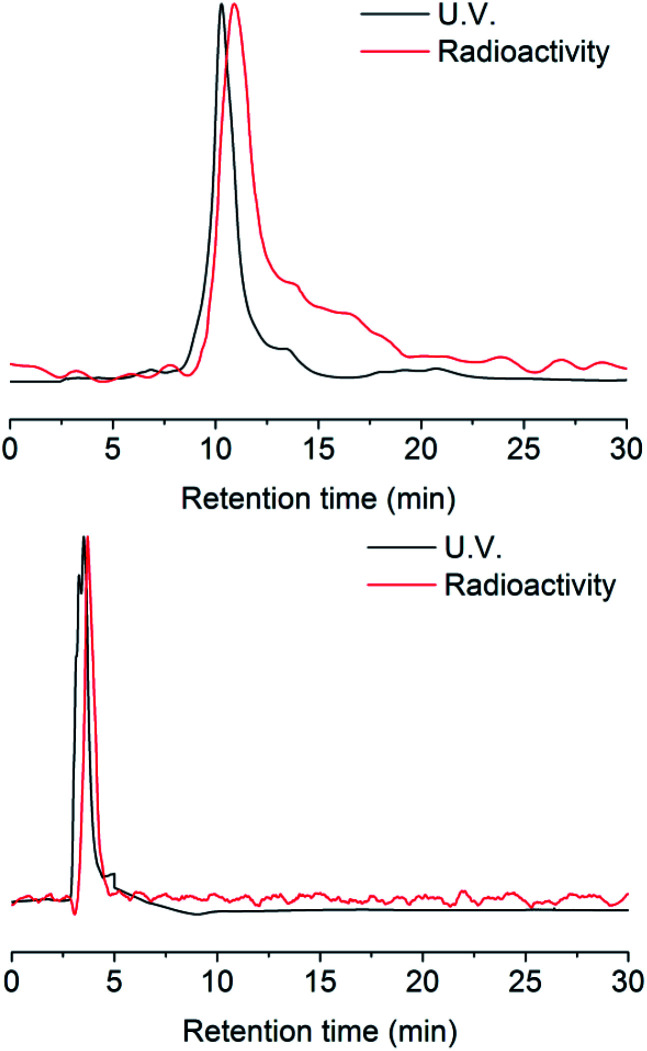
HPLC chromatograms of [^127^I]/[^131^I]2 and [^127^I]/[^131^I]3.

Radio-HPLC analysis demonstrated that ^131^I was labeled successfully to compound 2 with radiochemical yield (RCY) 21%. The molar activity of [^131^I]2 is about 8.4 GBq/μM (Fig. 1). In the previous work,^4^ we have already verified that even as little as radiolabeled compounds, DHP could react with NO specifically. Herein, the radio-TLC analysis was used to detect the cleavage reaction after stimulation by NO (Fig. S1†), which demonstrated [^131^I]3 could be split by NO into two fractions. The gold nanoparticle is one of the most widely studied and used nanoparticles with many mature surface modification methods developed,^22–24^ therefore we modify Pd@Au nanoplates with DHP for proof of concept.^25,26^ To prevent the hot molecules from being adsorbed by nanoplates, functionalized polyethylene glycol (NHS-PEG-SH) was employed to conjugate NHS onto nanoplates. The free PEG was removed by ultrafiltration centrifugation. After then, the NHS parts reacted with the amino group on compound 3, so that the radioactive ^131^I was linked to the nanoplates by DHP ([^131^I]4). Ultrafiltration centrifugation was used to remove the unattached radiolabeled molecules.

**Fig. 2:**
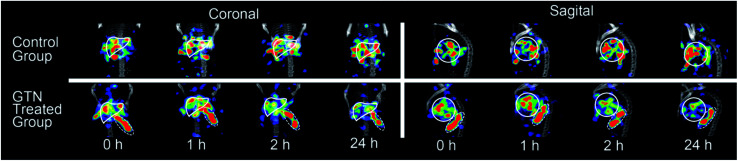
SPECT-CT images of mice livers (solid white lines) with [^131^I]4 after i.p. injection of saline (as control) and GTN.

MicroSPECT-CT imaging showed in Fig. 2 was employed for monitoring if the radio-modified nanoplates could response to NO *in vivo*. From previous literature, almost all the nanoparticles would become enriched in the liver 24 hours post injection.^25,26^ So, the time point of NO interventions was performed at 24 hours. GTN was chosen as the NO donor for cleavage triggering, according to previous literature in which the metabolism of GTN *in vivo* has been studied very thoroughly.^27^ After i.p. injection of GTN, the NO concentration in liver, heart and red blood cell were significantly elevated and reached the concentration required for the DHP cleavage. At the beginning, it was assumed that after the injection of GTN, the radioactivity uptake of the liver will decrease due to cleavage of the radionuclide part from the nanoplates. However, from the imaging, 1 hour post i.p. injection of GTN, the radioactivity of the liver increased. From the second hour post injection of GTN, the radioactivity of the liver began to decrease. As to the control group, there was no significant change in the radioactivity uptake of the liver in the first two hours post injection of normal saline. After 24 h injection of GTN, the radioactivity of the liver was significantly reduced. From the images, it could be concluded that the GTN treated group has fewer radioactivity residues than that in the control group.

**Fig. 3:**
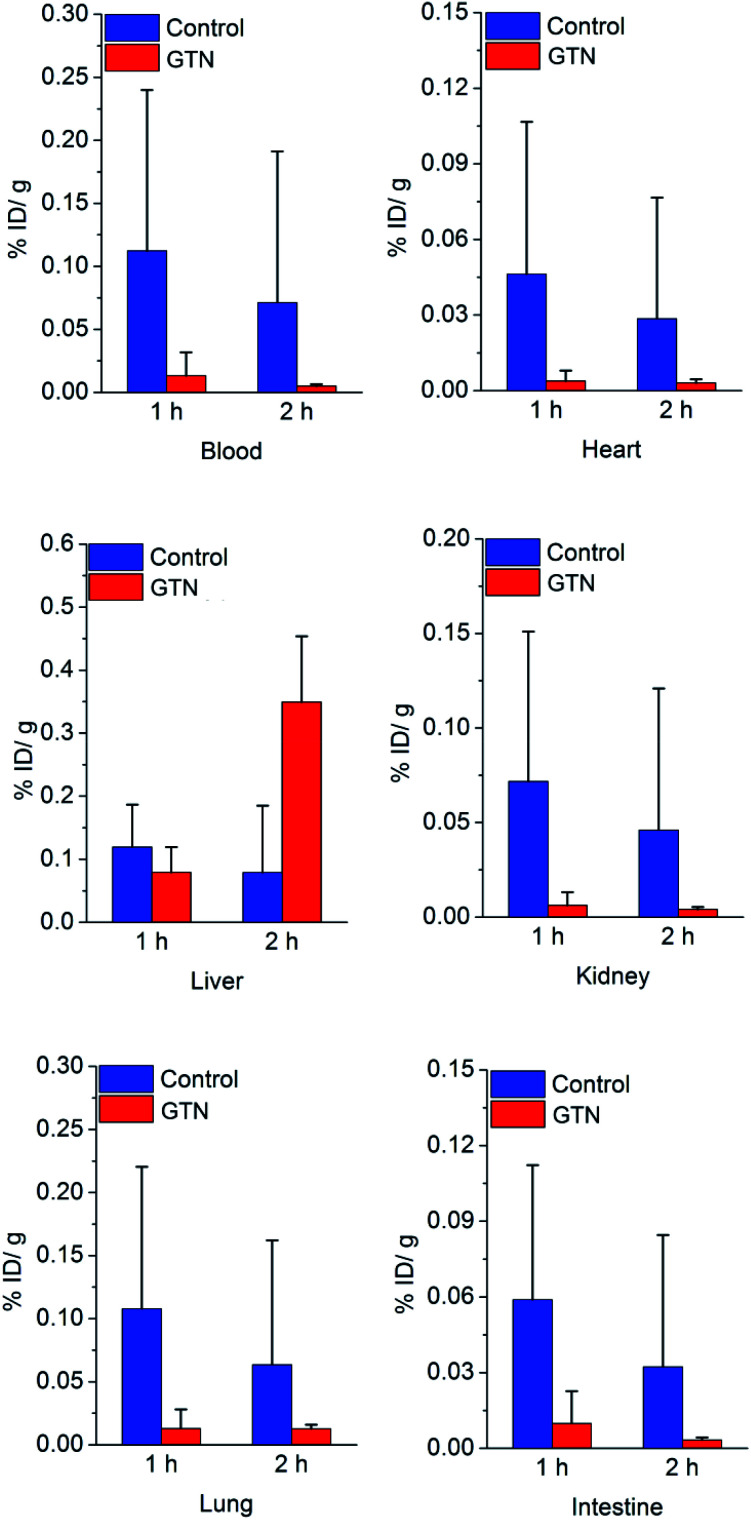
Biodistribution result of [^131^I]4 in mice after saline (as control) or GTN treatment, *P* < 0.05, with statistical difference.

We were still very curious about what happened in the experimental group in the first 2 hours post injection of GTN. Biodistribution study was performed to figure out what factors contribute to the increased uptake in the liver. Significant differences were showed between GTN treated group and the control group in the first hour post injection showed in Fig. 3.

The decreases of liver uptake were occurred once, which matches our expectation. In the second hour post injection, the liver uptake in GTN treated group significantly increased. These data confirm the results of imaging. Due to the first-pass metabolism, plasma, red blood cell (RBC), liver and heart are important sites for the metabolism of GTN. From the previous literature, we know that there are tens to hundreds of μmol rises in NO concentrations in RBC, heart, kidney post i.p. GTN injection.^27^ In our previous work, we have already demonstrated that the DHP will cleave at nmol level, which means the NO concentration post GTN injection is high enough to trigger the DHP cleave in these organs. The radioactivity in heart, blood, kidney in the control group was 12.1 times, 8.5 times, and 11.5 times that of the GTN treated group after 1 h injection of saline and GTN respectively. Other sufficient blood supply organs, such as lung and intestine had the same process as in liver and heart. The radioactivity in the intestine, lung of the control group was 8.3 times and 6.0 times that of the GTN treated group. The radioactivity in the heart, blood, kidney, intestine and lung of the control group was 9.1 times, 14.4 times, 11.1 times, 9.9 times, 5.0 times that of the GTN treated group after 2 h injection of saline and GTN respectively. After treated with GTN, the metabolism of the cut-off part with radionuclide has the same metabolic behavior. The cut-off parts cleaved from the nanoplates in other organs brought about the increased liver uptake 2 h post GTN injection. The cut-off part distribution caused the radionuclide secondary distribution. The biodistribution of cut-off part was demonstrated that this part has a typical behavior as lipophilic small molecules (Fig. S2†), high liver uptake and high blood uptake. We suppose the reason why there is a time gap between biodistribution data and imaging is that SPECT scanning process lasted 50 min, while the biodistribution data were collected on precise time points.

To validate the clearance of radionuclide indeed reduce the radiation dose loading. Organ dosimetry values were calculated by using organ level internal dose assessment software (OLINDA, version 1.1, Vanderbilt University).^28^ Biodistribution data of three time points were used to get the dosimetry values of all organs. In biodistribution study, organs like blood, heart, lung, kidney, and intestine showed a significant decrease of radioactivity uptake after treated with GTN, while these organs received a less internal dose. The internal dose that heart wall, thymus, lung, kidney receive in the control group was 10.6 times, 11.8 times, 3.4 times and 2.0 times that of the GTN treated group respectively. From Table 1, the radiation dose in the experimental group of which organs in the upper part of the torso received is much lower than that in the control group. This result may attribute to the clearance of radionuclides from heart and lung (because the software is modeled on human, some organs (breast) of the mouse that are not in the upper part of the torso may cause deviation in the data). The internal dose that total body received in the control group was 2.1 times that of the GTN treated group, which demonstrated this strategy has an obvious effect on clearance of radionuclides and could reduce the radiation dose loading effectively.

**Table 1:** Portion of sample output from OLINDA 1.1

Target organ	Mean absorbed radiation dose (mS V^−1^/MBq)		
	Control group	GTN treated group	GTN/Ctrl
Adrenals	8.65 × 10^−5^	4.82 × 10^−5^	56%
Brain	2.33 × 10^−6^	1.19 × 10^−6^	51%
Breasts	4.42 × 10^−5^	8.76 × 10^−6^	20%
Gallbladder wall	5.20 × 10^−5^	3.99 × 10^−5^	77%
LLI wall	1.24 × 10^−5^	6.81 × 10^−6^	55%
Small intestine	2.86 × 10^−5^	1.63 × 10^−5^	57%
Stomach wall	2.09 × 10^−4^	7.26 × 10^−5^	35%
ULI wall	3.27 × 10^−5^	1.87 × 10^−5^	57%
Heart wall	4.05 × 10^−4^	3.51 × 10^−5^	9%
Kidneys	4.02 × 10^−4^	1.97 × 10^−4^	49%
Liver	1.73 × 10^−4^	2.56 × 10^−4^	148%
Lungs	2.21 × 10^−4^	6.46 × 10^−5^	29%
Muscle	2.71 × 10^−5^	1.35 × 10^−5^	50%
Ovaries	1.38 × 10^−5^	7.86 × 10^−6^	57%
Pancreas	1.65 × 10^−4^	1.01 × 10^−4^	61%
Red marrow	3.19 × 10^−5^	1.32 × 10^−5^	41%
Osteogenic cell	2.64 × 10^−5^	1.14 × 10^−5^	43%
Skin	1.19 × 10^−5^	5.46 × 10^−6^	46%
Spleen	4.26 × 10^−3^	4.23 × 10^−3^	99%
Testes	1.64 × 10^−6^	1.28 × 10^−6^	78%
Thymus	1.20 × 10^−4^	1.02 × 10^−5^	9%
Thyroid	9.70 × 10^−6^	2.70 × 10^−6^	28%
Urinary bladder	0.00 × 10^−1^	0.00 × 10^−1^	N/A
Uterus	1.21 × 10^−5^	6.79 × 10^−6^	56%
Total body	3.10 × 10^−5^	1.49 × 10^−5^	48%

This strategy can be also used to minimize the cycle of tracers screen. In general, the most time-consuming and critical step in the development of new radiotracers is to screen compounds with different substituents of the same backbone structures to look for those with desirable pharmacokinetic and metabolic properties. Even when the radiotracers are thoroughly screened and optimized, the non-specific binding is evitable. The strategy can decrease uptake of blood and liver is efficient to increase the contrast, which offers chances for the tracers mediocre in imaging, but cheap in price.

## Conclusions

In summary, a “turn off” strategy of controllable clearance of radionuclide from nanoparticle was established for the first time. ^131^I was labeled on 1,4-dihydropyridine that was decorated on gold nanoplates. A cleavage between radionuclide and nanoparticle was validated both in aqueous solutions and *in vivo* after treated with NO. MicroSPECT imaging and biodistribution assay validated the clearance of radionuclide from the nanoparticle. Dosimetry calculation betrayed a significant decrease of radiation dose in GTN treated group. The clearance from blood and liver was an effective way to increase the contrast. This strategy is applicable to other long half-life radionuclides such as ^64^Cu, ^111^In, ^124^I, and provides the possibility of multi-tracer imaging.

## Conflicts of interest

There are no conflicts to declare.

## Supplementary Material

RA-010-D0RA07186B-s001
