# Supplementary material for: Controllable stripping of radiolabeled group in vivo to optimize nuclear imaging via NO-responsive bioorthogonal cleavage reaction
Source: RSC Adv. 2020 Nov 3;10(66):40030–4. doi: 10.1039/d0ra07186b (PMC9057491; doi:10.1039/d0ra07186b)
Supplement: RA-010-D0RA07186B-s001 [file RA-010-D0RA07186B-s001.pdf]

Electronic Supplementary Material (ESI) for RSC Advances.
This journal is © The Royal Society of Chemistry 2020

Supporting Information for

Controllable stripping of radiolabeled group *in vivo* to optimize nuclear imaging via NO-responsive bioorthogonal cleavage reaction

Li *et al.*

Table of content

Synthesis and methods........1
Experiments and characterization........2
Radiolabeling conditions........3
Verification of cleavage in solution........4
Modification of nanoplates........4
SPECT-CT imaging in mice........5
Biodistribution in mice........5
Time points of injection and sacrifice........5
Biodistribution of cut-off part........5
Reference........6

Synthesis and methods

Reagents and solvents were purchased from commercial resources and used without further purification. The ^{1}H NMR spectra and ^{13}C NMR were obtained on a Bruker spectrometer (Avance III 600 MHz, USA). Mass spectrometry was acquired from a Bruker Equire 3000 plus (ESI) instrument (USA). High-resolution mass spectrometry (HRMS) was acquired from a Q Exactive LC-MS/MS system (Waters, USA). HPLC analysis was achieved on a HYPERSIL GOLD C18 coloum (Thermo Scientific, 5 μm, 10 mm × 250 mm). SPECT/CT imaging was performed using a pinhole collimator microSPECT/CT scanner (Mediso, Budapest, Hungary). The activitied of organs and tissues harvest from mice was measured by a gamma counter (WIZARD 2480, Perkin-Elmer, Downers Grove, IL, USA). The cleavage of compound [^{131}I]**3** was detected by

a Mini-Scan radio-TLC Scanner (BioScan, Poway, CA, USA).

Experiments and characterization

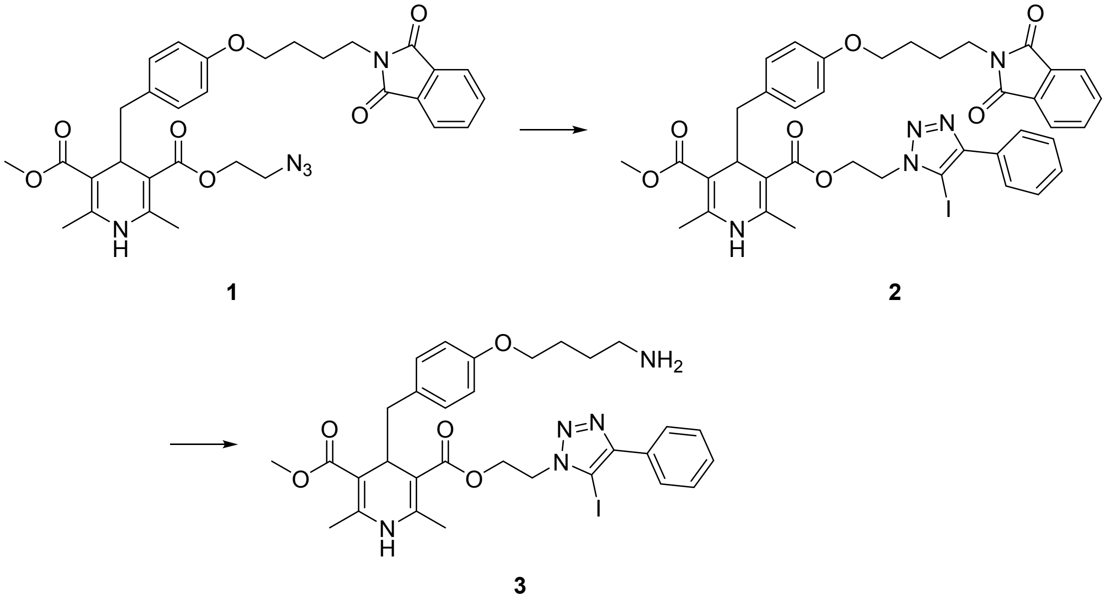

Scheme S1. Synthetic route of standard compounds.

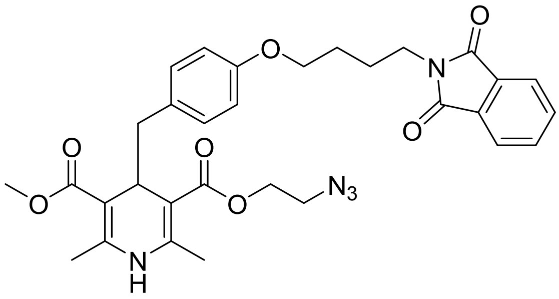

3-(2-azidoethyl) 5-methyl 4-(4-(4-(1,3-dioxoisoindolin-2-yl)butoxy)benzyl)-2,6-dimethyl-1,4-dihydropyridine-3,5-dicarboxylate (1)

1 was synthesized following a previous literature.[1]

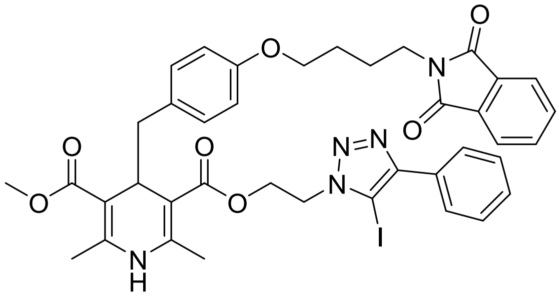

3-(2-(5-iodo-4-phenyl-1H-1,2,3-triazol-1-yl)ethyl) 5-methyl 4-(4-(4-(1,3-

dioxoisoindolin-2-yl)butoxy)benzyl)-2,6-dimethyl-1,4-dihydropyridine-3,5-dicarboxylate

A mixture of **1** (0.59 g, 1 mmol), (iodoethynyl)benzene (0.23 g, 1 mmol), CuI (10 mg, 0.05 mmol) and triethylamine (0.20 g, 2 mmol) in CH_2Cl_2 (10 mL) was stirred overnight at room temperature. The solvent was then evaporated in vacuo to give the residue which was purified by column chromatography (silica gel, petroleum ether/ethyl acetate 2:1) to give a white solid (0.19 g, 0.23 mmol, 23%). ^{1}H NMR ($CDCl_3$, 400 MHz) δ 7.97 (2H, d, J = 7.4 Hz), 7.87-7.85 (3H, m), 7.76-7.74 (2H, m), 7.49 (2H, t, J = 7.6 Hz), 6.86 (2H, d, J = 8.4 Hz), 6.71 (2H, d, J = 8.4 Hz), 4.92-4.86, (1H, m), 4.77 (2H, t, 5.4 Hz), 4.70-4.65 (1H, m), 4.52-4.47 (1H, m), 4.00 (2H, t, J = 5.7 Hz), 3.77 (2H, t, J = 6.8 Hz), 3.63 (3H, s), 2.17 (3H, s), 2.12 (3H, s), 1.94-1.82 (4H, m). ^{13}C NMR ($CDCl_3$, 100 MHz) δ 186.4, 186.1, 167.1, 157.1, 149.8, 147.1, 145.7, 134.0, 132.1, 131.1, 131.0, 130.3, 128.5, 127.5, 123.2, 113.3, 101.6, 100.2, 67.0, 61.4, 53.4, 50.8, 49.6, 41.1, 37.7, 35.2, 29.7, 26.5, 26.4, 25.3, 19.3, 18.9. HRMS (FTMS ESI$^+$): m/z calcd for $C_{39}H_{38}IN_5NaO_7^+$ ([M + Na]$^+$) 838.1714; found 838.0174.

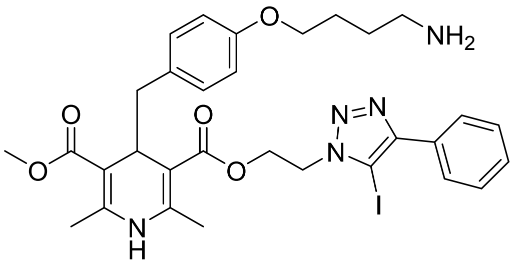

3-(2-(5-iodo-4-phenyl-1H-1,2,3-triazol-1-yl)ethyl) 5-methyl 4-(4-(4-aminobutoxy)benzyl)-2,6-dimethyl-1,4-dihydropyridine-3,5-dicarboxylate

A mixture of **2** (0.19 g, 0.23 mmol), hydrazinium hydroxide (0.05 mL) in ethanol (5 mL) was stirred for 10 h at room temperature. The solution was then basified with KOH solution (1 M, 10 mL), and extracted with CH_2Cl_2 (10 mL × 3) before the organic layer was dried with anhydrous Na_2SO_4. The solvent was evaporated in vacuo to give the residue (0.85 g, 92%) which was directly used in the next step without further purification. HRMS(FTMS ESI$^+$): m/z calcd for $C_{31}H_{37}IN_5O_5^+$ ([M + H]$^+$), 686.1839; found 686.2369.

Radiolabeling conditions

3-(2-(5-(iodo-^{131}I)-4-phenyl-1H-1,2,3-triazol-1-yl)ethyl) 5-methyl 4-(4-(4-(1,3-dioxoisoindolin-2-yl)butoxy)benzyl)-2,6-dimethyl-1,4-dihydropyridine-3,5-dicarboxylate

The $CuCl_2$/TEA complex solution was prepared by following the previous literature. In a centrifuge tube (1.5 mL), (iodoethynyl)benzene (1 μmol) was added into the $CuCl_2$/TEA complex solution (40 μL). After 5 min, Na[^{131}I]I (5 mCi) in water (10 μL) was added to the solution above. Saturated acetonitrile solution of compound 1 (80 μL) was added to the mixture above. The tube was heated at 60 ^{o}C for 90 min. The reaction mixture was diluted with acetonitrile, the resulting solution was purified by HPLC. The solvent of the collection was evaporated under a stream of nitrogen at 90 ^{o}C.

3-(2-(5-(iodo-^{131}I)-4-phenyl-1H-1,2,3-triazol-1-yl)ethyl) 5-methyl 4-(4-(4-aminobutoxy)benzyl)-2,6-dimethyl-1,4-dihydropyridine-3,5-dicarboxylate

[^{131}I]**1** was dissolved in ethanol (1 mL), 80% hydrazinium hydroxide (20 μL) was added. The vial was shook for 2 h. The solvent of the collection was evaporated under a stream of nitrogen at 50 ^{o}C.

Verification of cleavage in solution

In a centrifuge tube, [^{131}I]**3** (100 mCi) was dissolved in ethanol (1 mL), NO saturated water solution (20 μL) was added. Vortex mixing was used. Radio-TLC was used to monitor the cleavage.

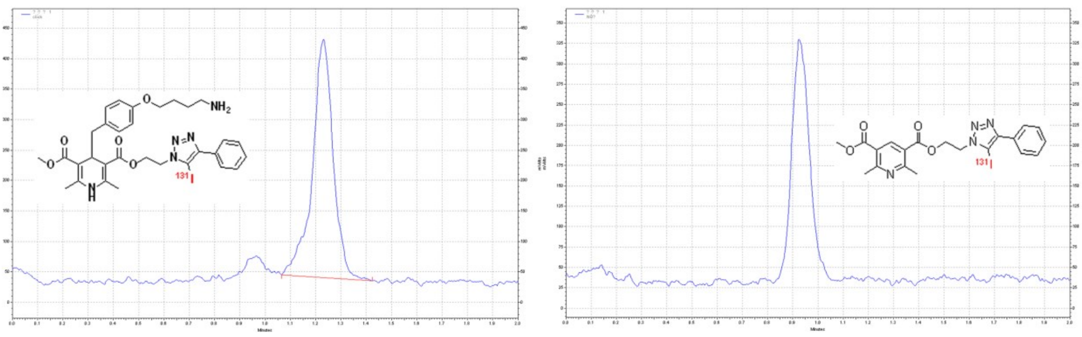

Figure S1. Radio-TLC spectrum of [^{131}I]**3** (left) and [^{131}I]**3** treated with NO (right).

Modification of nanoplates

Nanoplates (1.0 mg) were precipitated with acetone and re-dispersed in HS-PEG-NHS aqueous solution (20 mg mL^{-1}, 1.0 mL). The mixture was kept in a refrigerator overnight. Ultrafiltration was used to remove free PEG.

The resulting nanoplates were re-dispersed in [^{131}I]**3** aqueous solution (1.0 mL). The mixture was kept in a refrigerator overnight. Ultrafiltration was used to remove free [^{131}I]**3**.

SPECT-CT imaging in mice

All animal protocols were approved by the Institute's Animal Care and Use Committee. About 200 μCi radiolabelled nanoplates were injected to normal male BALB/c mice which were anesthetized with isoflurane.

Biodistribution in mice

Biodistribution was performed on Balb/c mice. [^{131}I]**4** was administered into each mouse through tail vein injection. The mice were sacrificed at different time points. Radioactivities of major organs was measured by gamma counter. The results were shown as a percentage of the injected dose per gram of tissue (%ID/g).

Time points of injection and sacrifice

0 h, [^{131}I]**4** was injected into each mouse.

24 h, GTN was injected to mice in the experiment group; saline was injected to mice in the control group.

25 h, mice were sacrificed to get the data which were performed as 1 h post injection.

26 h, mice were sacrificed to get the data which were performed as 2 h post injection.

Biodistribution of cut-off part

Biodistribution was performed on Balb/c mice. Cut-off part was administered into each

mouse through tail vein injection. The mice were sacrificed at different time points. Radioactivities of major organs was measured by gamma counter. The results were shown as a percentage of the injected dose per gram of tissue (%ID/g).

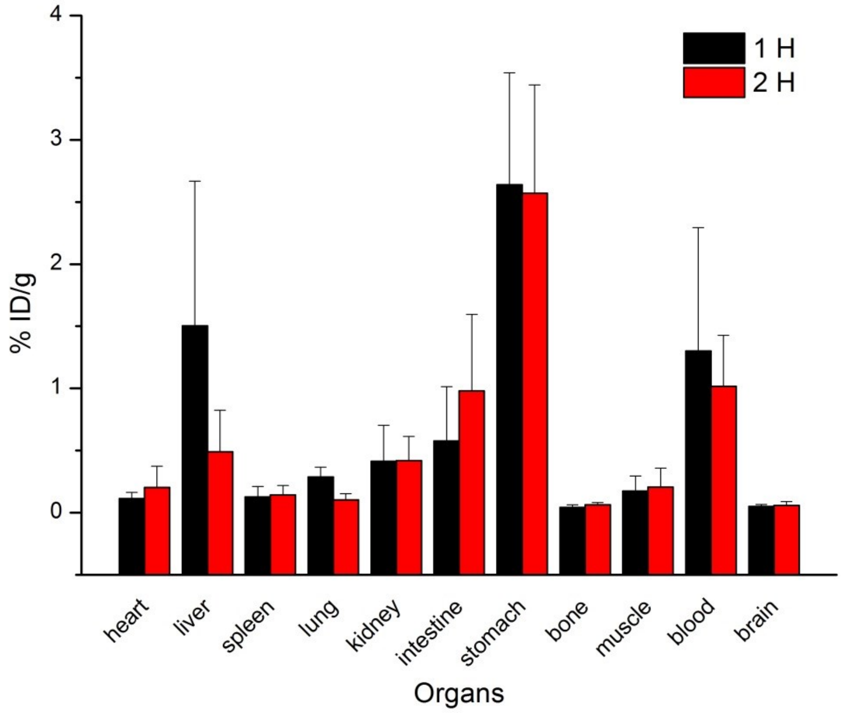

Figure S2. Biodistribution result of cut-off part in mice.

Reference

[1] H. Li, D. Zhang, M. Gao, L. Huang, L. Tang, Z. Li, X. Chen, X. J. C. s. Zhang, **2017**, *8*, 2199-2203.
